# Supplementary material for: Silibinin Suppresses Tumor Cell-Intrinsic Resistance to Nintedanib and Enhances Its Clinical Activity in Lung Cancer
Source: Cancers (Basel). 2021 Aug 19;13(16):4168. doi: 10.3390/cancers13164168 (PMC8394850; doi:10.3390/cancers13164168)
Supplement: Supplementary file 1 [file cancers-13-04168-s001.zip › cancers-1318677-original-images.pdf]

Silibinin suppresses tumor cell-intrinsic resistance to nintedanib and enhances its clinical activity in lung cancer

Uncropped images

Figure 3 NTD-responsive 24h

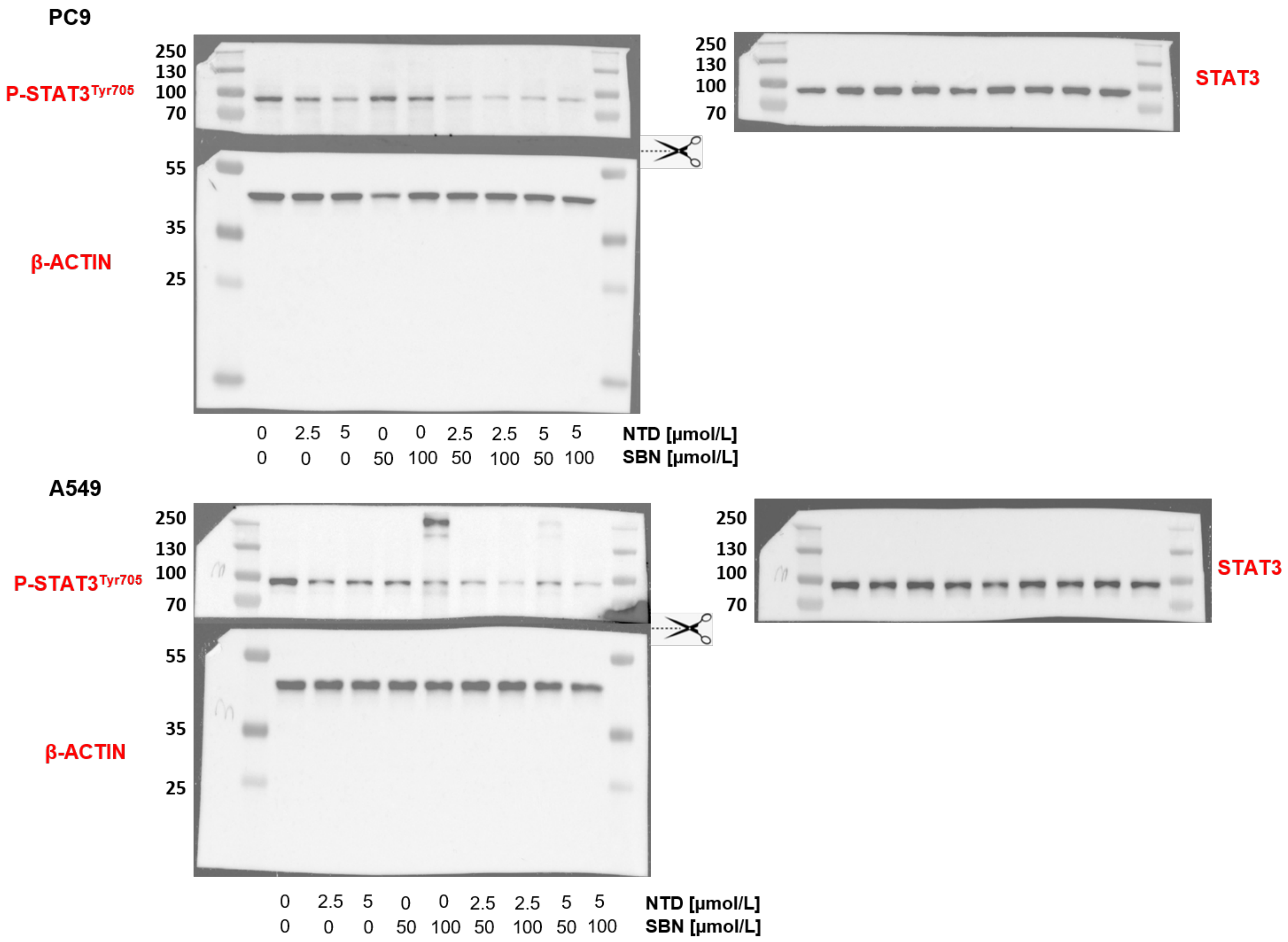

Figure 3 NTD-resistant 24h

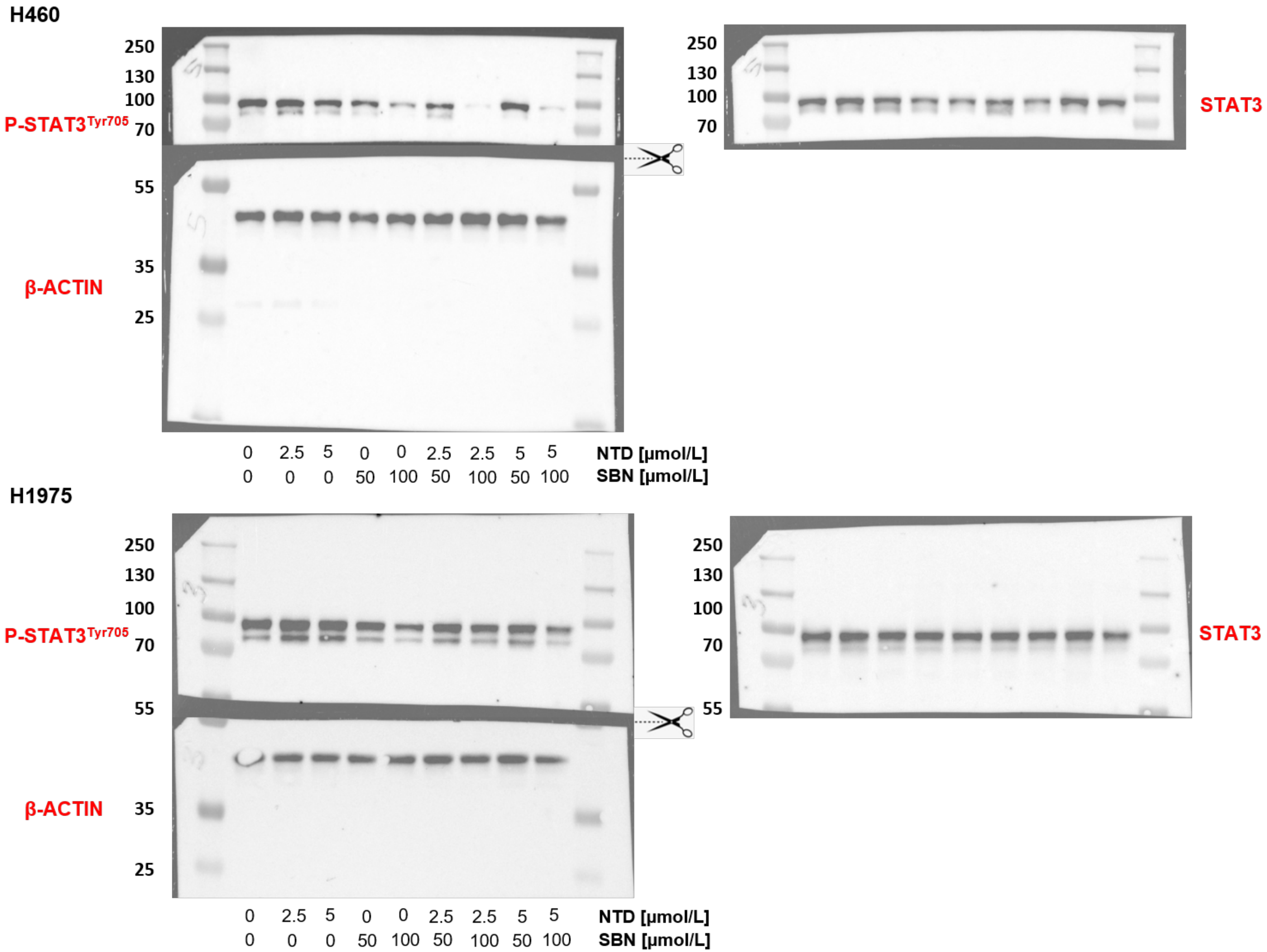

Figure 3 NTD-resistant 48h

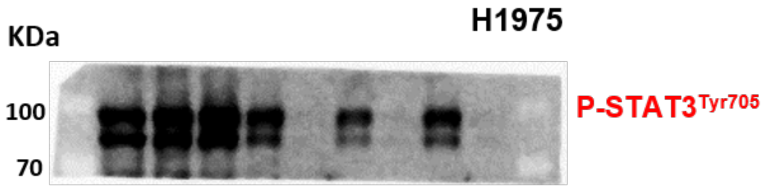

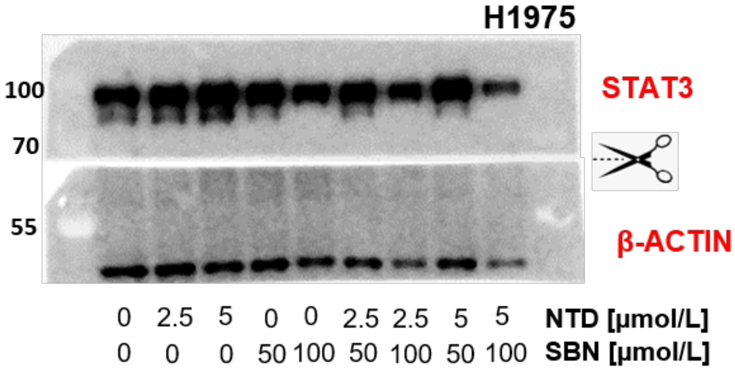

Figure S3 A Broken boxes marks the final cropped images

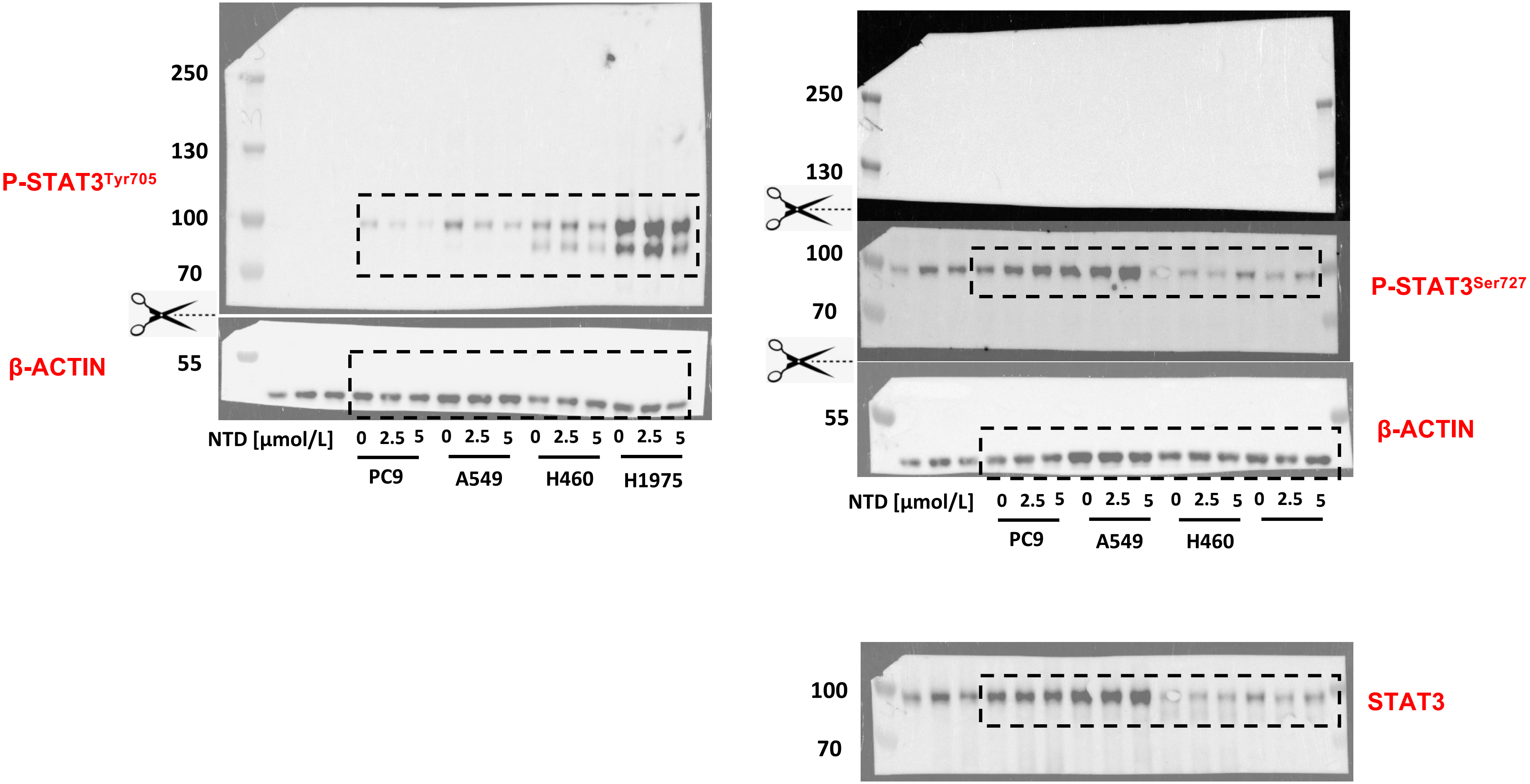

Figure S3 B

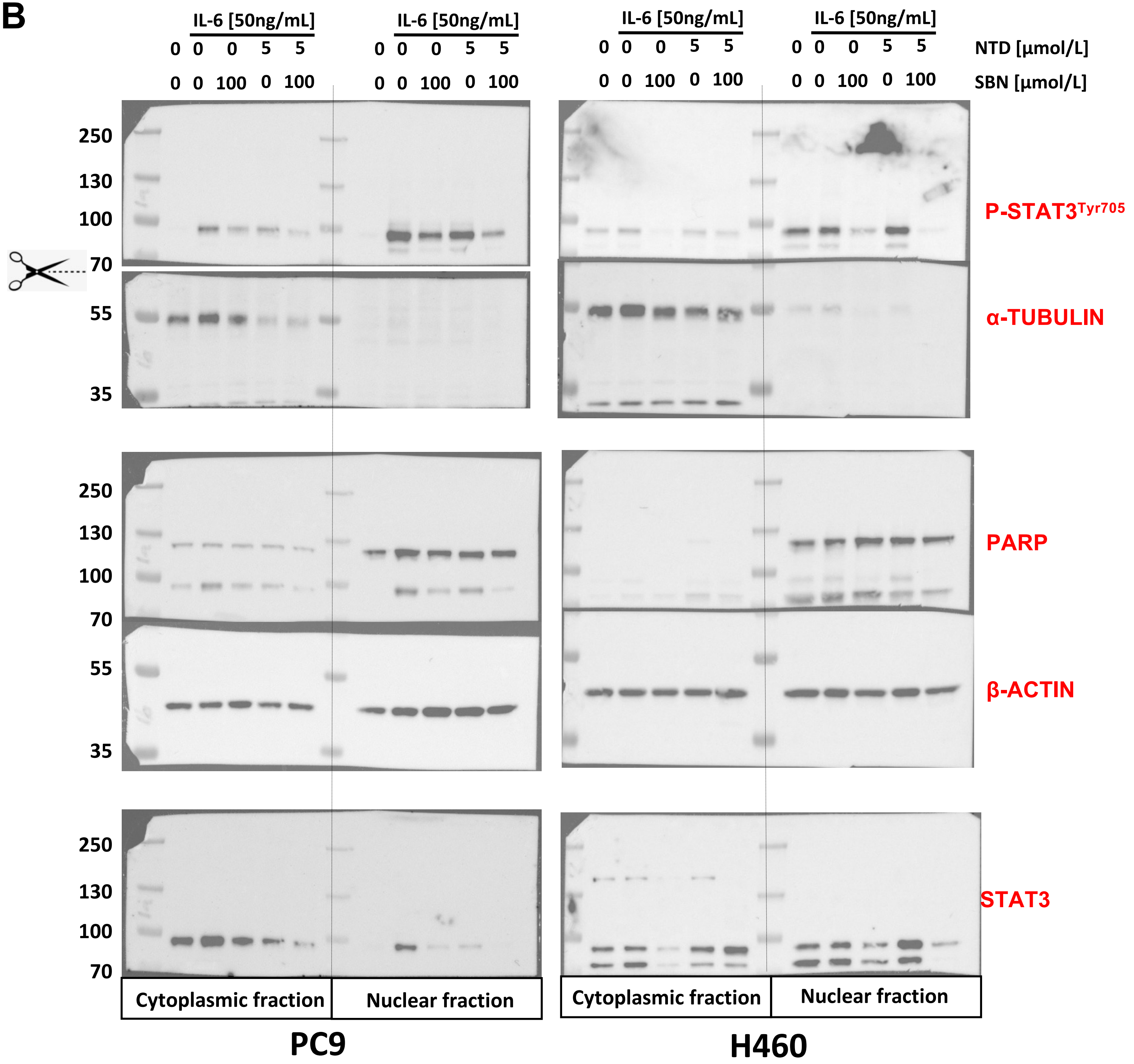

IL-6 [50ng/mL]
IL-6 [50ng/mL]
IL-6 [50ng/mL]
IL-6 [50ng/mL]
0 0 0 5 5
0 0 100 0 100
NTD [µmol/L]
SBN [µmol/L]
250
130
100
70
55
35
P-STAT3Tyr705
α-TUBULIN
PARP
β-ACTIN
STAT3
Cytoplasmic fraction
Nuclear fraction
Cytoplasmic fraction
Nuclear fraction
PC9
H460
