# Supplementary material for: Silibinin Suppresses Tumor Cell-Intrinsic Resistance to Nintedanib and Enhances Its Clinical Activity in Lung Cancer
Source: Cancers (Basel). 2021 Aug 19;13(16):4168. doi: 10.3390/cancers13164168 (PMC8394850; doi:10.3390/cancers13164168)
Supplement: Supplementary file 1 [file cancers-13-04168-s001.zip › cancers-1318677-suppl-final.pdf]

Supplementary Materials: Silibinin Suppresses Tumor Cell-Intrinsic Resistance to Nintedanib and Enhances Its Clinical Activity in Lung Cancer

Joaquim Bosch-Barrera, Sara Verdura, José Carlos Ruffinelli, Enric Carcereny, Elia Sais, Elisabet Cuyàs, Ramon Palmero, Eugeni López-Bonet, Alejandro Hernández-Martínez, Gloria Oliveras, Maria Buxó, Angel Izquierdo, Teresa Morán, Ernest Nadal and Javier A. Menéndez

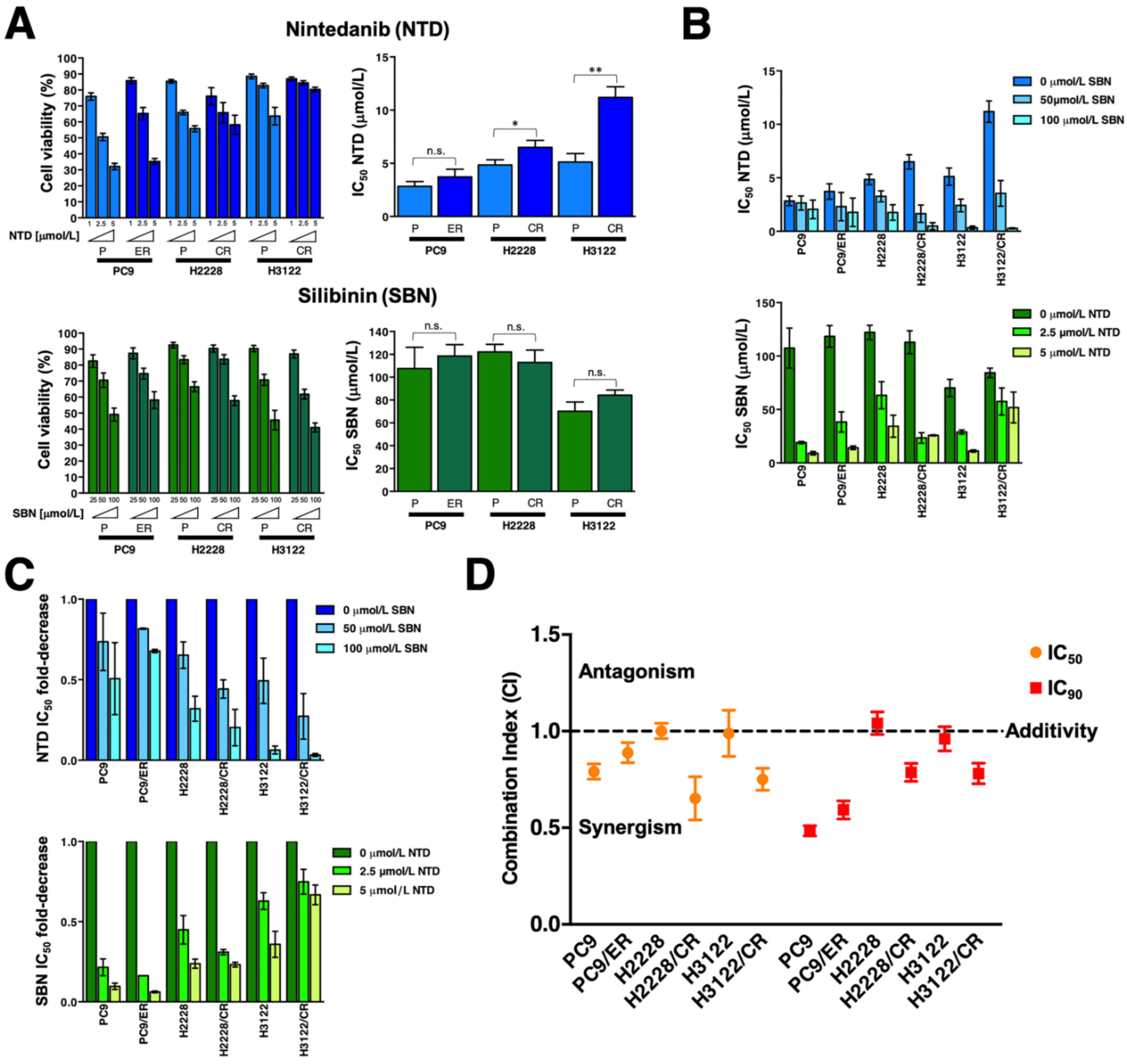

Figure S1. Analysis of the cytotoxic interaction between nintedanib and silibinin in NSCLC cells with acquired resistance to erlotinib and crizotinib. A. *Left panels.* The metabolic status of parental (P) PC-9, H2228, and H3122 cell lines and erlotinib- and crizotinib- (ER and CR) resistant derivatives treated with increasing concentrations of nintedanib (NTD; 1.25, 2.5, and 5 µmol/L) and Scheme 25. 50, and 100 µmol/L) was measured using MTT uptake assays, and cell viability is expressed as % uptake relative to untreated control cells (=100% cell viability). *Right panels.* Bar graphs of the IC_{50} values for each cell line calculated from the MTT assays as described in "Materials and methods". The results are presented as the means (*columns*) ± S.D. (*bars*) (n=3, in triplicate). B. Bar graphs showing the MTT-based IC_{50} values of NTD (*top*) and SBN (*bottom*) for each cell line calculated in the absence or presence of graded concentrations of SBN and NTD, respectively. C. Bar graphs showing the fold-change in NTD (top) and SBN (bottom) IC_{50} values obtained in the absence or presence of graded concentrations of SBN and NTD, respectively. D. Computed combination index (CI) values for the

combination of NTD and SBN at 50% and 90% effect levels. CI values less than, equal to, or greater than 1 indicates synergy, additivity, or antagonism, respectively. The horizontal line at CI=1 is the line of additivity. The results in A, B, C, and D panels are presented as the means (*columns*) ± S.D. (*bars*) (n=3, in triplicate).

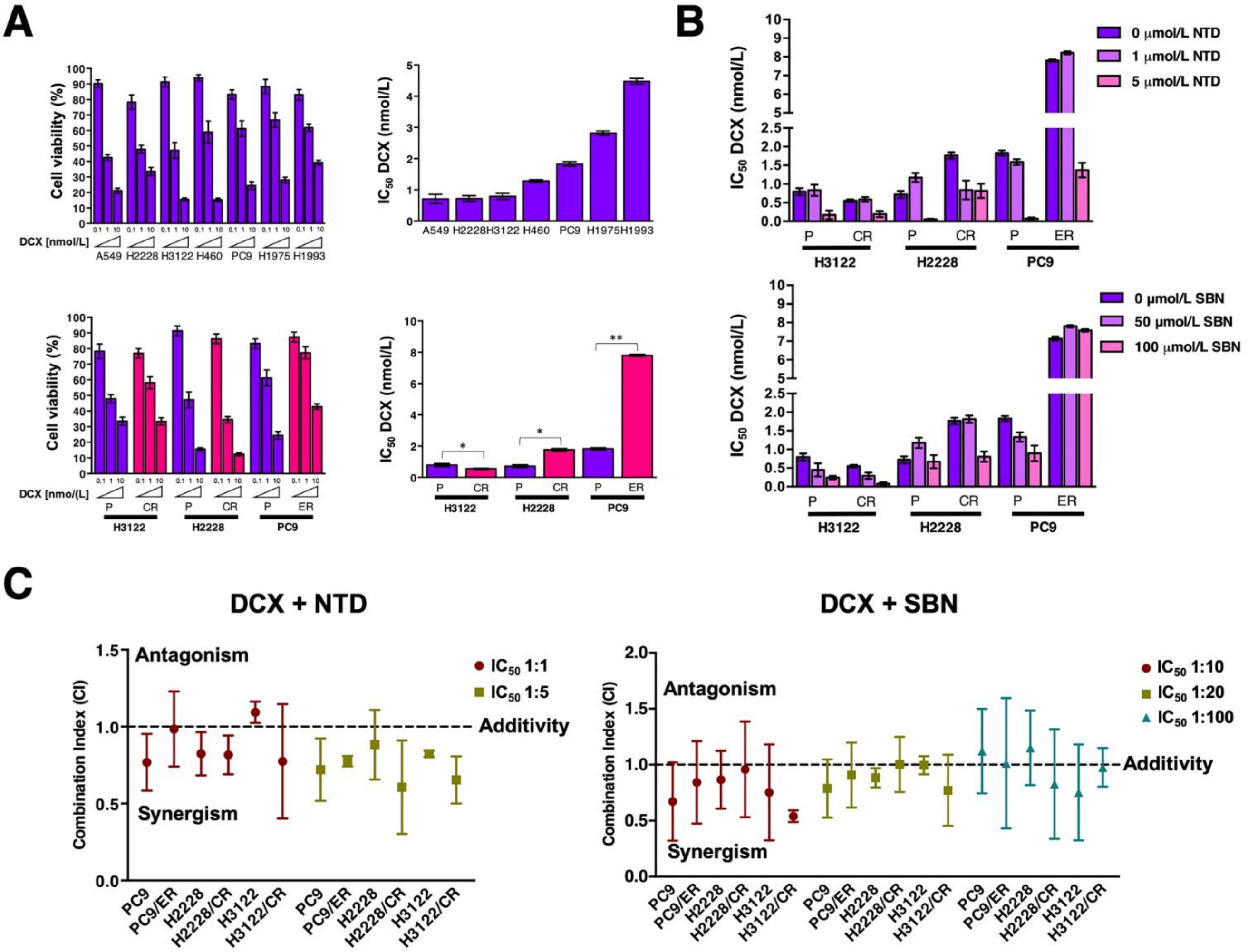

Figure S2. Analysis of the cytotoxic interaction between docetaxel, nintedanib, and silibinin in NSCLC cells with acquired resistance to erlotinib and crizotinib. A. *Left panels.* The metabolic status of parental (*top*) and erlotinib- and crizotinib- (ER and CR) resistant derivatives (*bottom*) treated with increasing concentrations of docetaxel (DCX; 0.1, 1.0, and 10 nmol/L) was measured using MTT uptake assays, and cell viability is expressed as % uptake relative to untreated control cells (=100% cell viability). *Right panels.* Bar graphs of the IC_{50} values for each cell line calculated from the MTT assays as described in "Materials and methods". The results are presented as the means (*columns*) ± S.D. (*bars*) (n=3, in triplicate). B. Bar graphs showing the MTT-based IC_{50} values of DCX for each pair of parental (P) and erlotinib- and crizotinib-resistant derivatives calculated in the absence or presence of graded concentrations of NTD (*top*) and SBN (*bottom*). C. Computed combination index (CI) values for the combination of DCX *plus* NTD (*left*) and DCX *plus* SBN (*right*) at 50% effect levels using different fixed ratio combinations of the drugs. CI values less than, equal to, or greater than 1 indicates synergy, additivity, or antagonism, respectively. The horizontal line at CI=1 is the line of additivity. The results in A, B, and C panels are presented as the means (*columns*) ± S.D. (*bars*) (n=3, in triplicate).

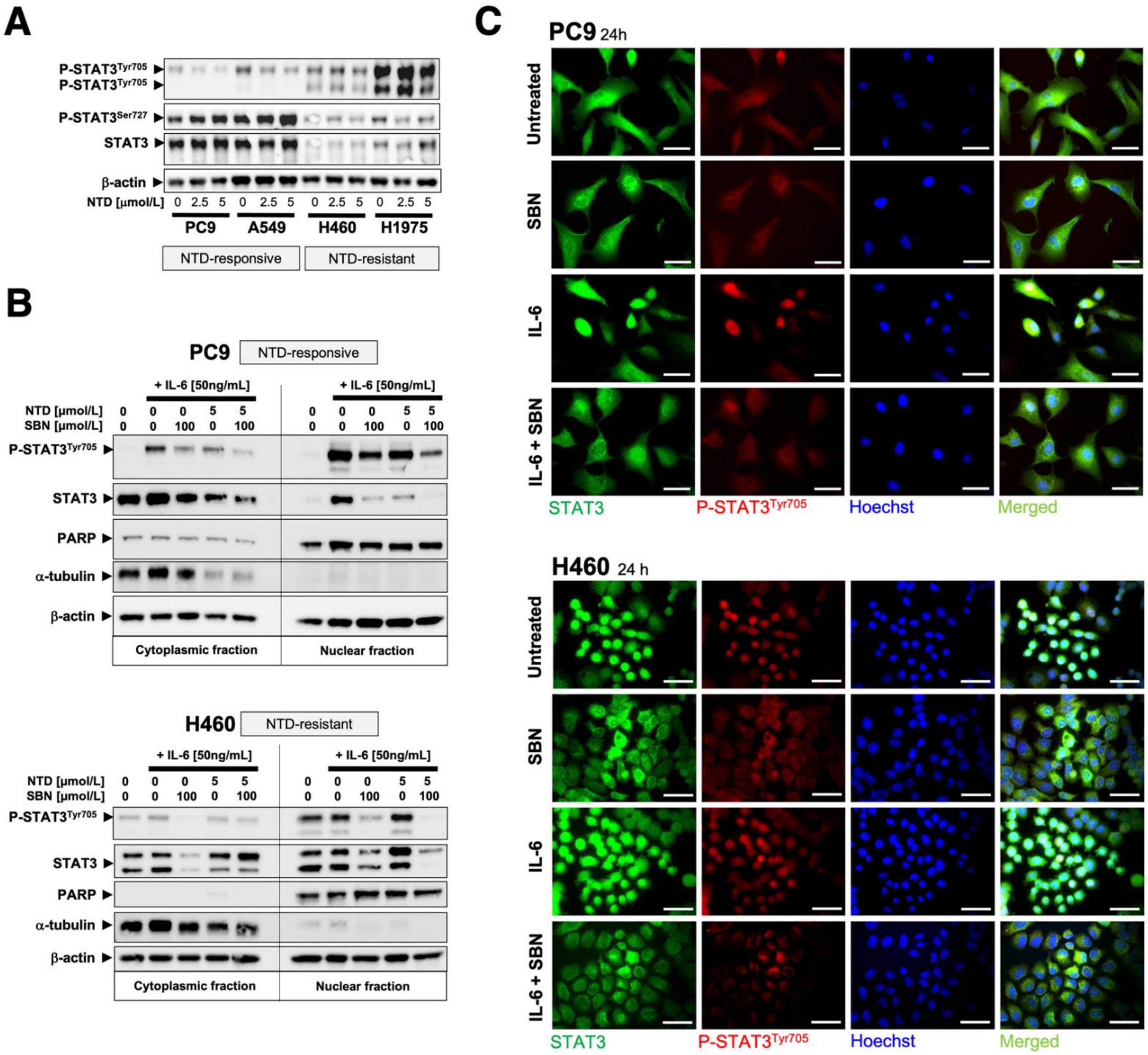

Figure S3. Effects of silibinin on the intracellular distribution of STAT3 and phospho-STAT3^{Tyr705}. **A.** Nintedanib (NTD)-responsive PC-9 and A549 cells, and NTD-resistant H460 and H1975 cells were serum-starved overnight and then left untreated or treated with graded concentrations of NTD for 24 h. Levels of phospho-STAT3^{Tyr705}, phospho-STAT3^{Ser727}, and STAT3 were detected by immunoblotting using specific antibodies. Figures show representative immunoblots of multiple ($n \geq 3$) independent experiments. **B.** Levels of phospho-STAT3^{Tyr705} and STAT3 were detected by immunoblotting in cytoplasmic and nuclear fractions of PC-9 (*top*), and H460 cells (*bottom*) cultured in the absence or presence NTD, SBN, and/or 50 ng/mL IL-6. Figures show representative immunoblots of multiple ($n \geq 3$) independent experiments. **C.** PC9 cells (*top*) and H460 cells (*bottom*) stimulated with IL-6 (50 ng/mL) in the absence or presence of silibinin (100 µmol/L). After 24 h, cells were fixed with ice-cold methanol and stained for total STAT3 or phospho-STAT3 Tyr705, followed by Alexa Fluor®-conjugated secondary antibody and Hoechst counterstaining. Figure shows representative immunofluorescence microphotographs of at least 3 independent experiments performed in triplicate. The scale bar indicates 50 µm.

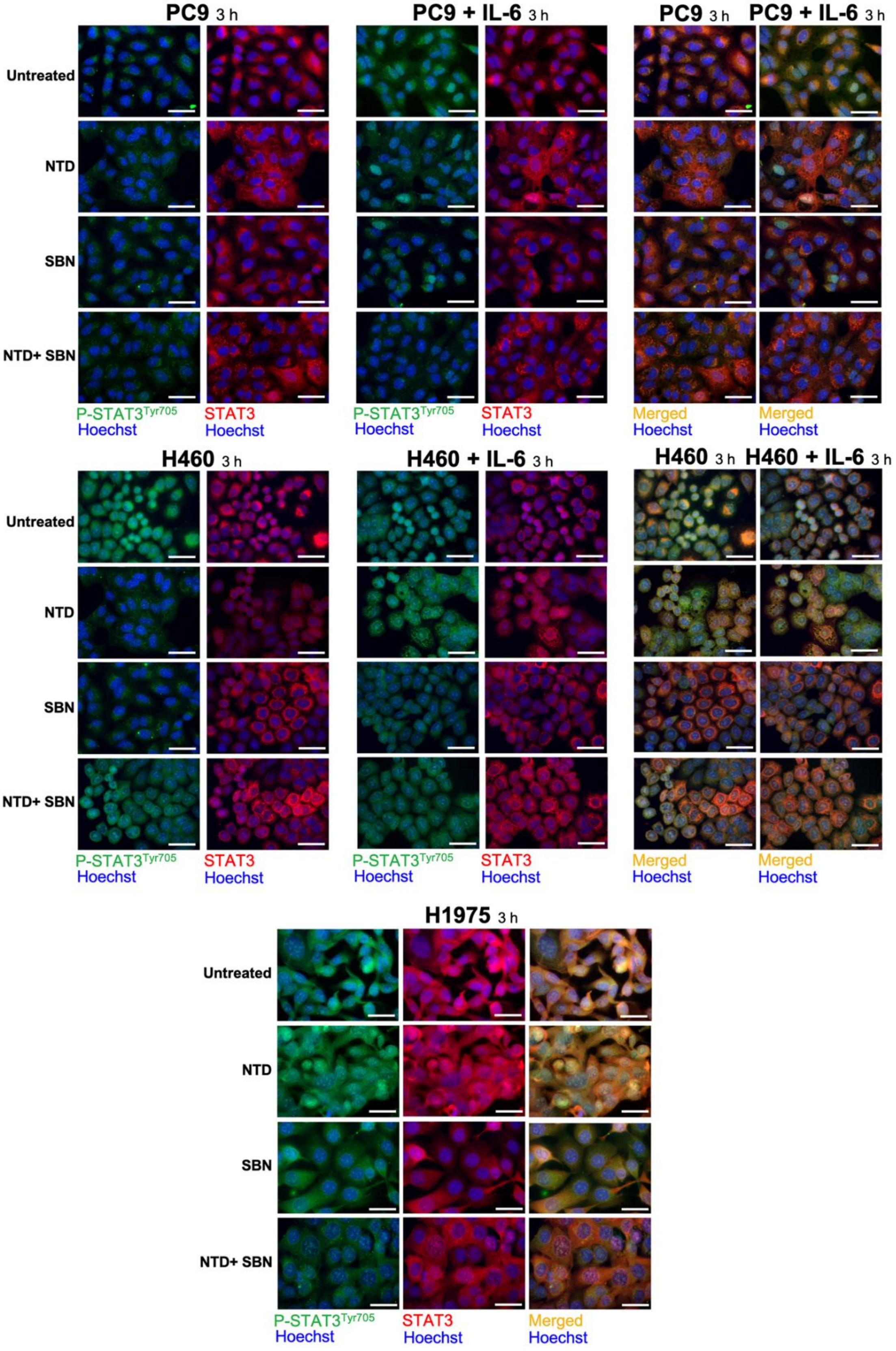

Figure S4. Effects of nintedanib and silibinin on the intracellular distribution of STAT3 and phospho-STAT3^{Tyr705}. PC9, H460, and H1975 cells (stimulated or not with IL-6, 50 ng/mL) were cultured in the absence or presence of nintedanib (NTD, 10 µmol/L) and/or silibinin (SBN, 100 µmol/L). After 3 h, cells were fixed with ice-cold methanol and stained for total STAT3 or phospho-STAT3 Tyr705, followed by Alexa Fluor®-conjugated secondary antibody and Hoechst counter-staining. Figure shows representative immunofluorescence microphotographs of at least 3 independent experiments performed in triplicate. The scale bar indicates 50 µm.

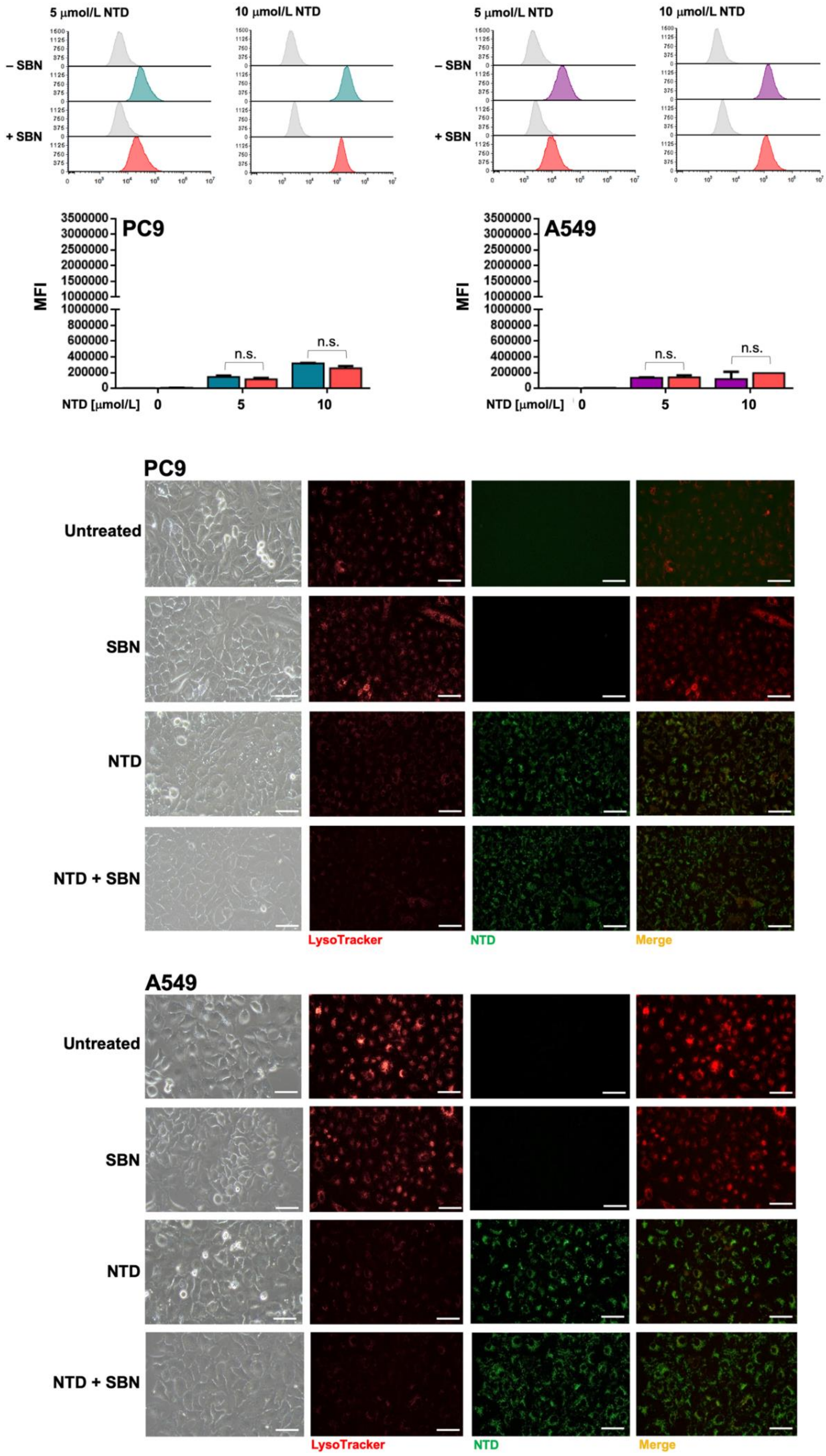

Figure S5. Effects of silibinin on the lysosomal sequestration of nintedanib in nintedanib-sensitive NSCLC tumor cells. The impact of 100 μmol/L silibinin (SBN; 1 h pre-treatment) on the intracellular accumulation of 5 and 10 μmol/L

nintedanib (NTD) in PC9 and A549 cells was analyzed by flow cytometry (*top panels*) and live cell fluorescence microscopy (*bottom panels*) after 3 h drug exposure. Each experimental value represents the mean NTD-associated fluorescence (*columns*) ± S.D. (*bars*) of 3 independent experiments. The scale bar indicates 50 μm. (MFI: Mean Fluorescence Intensity).

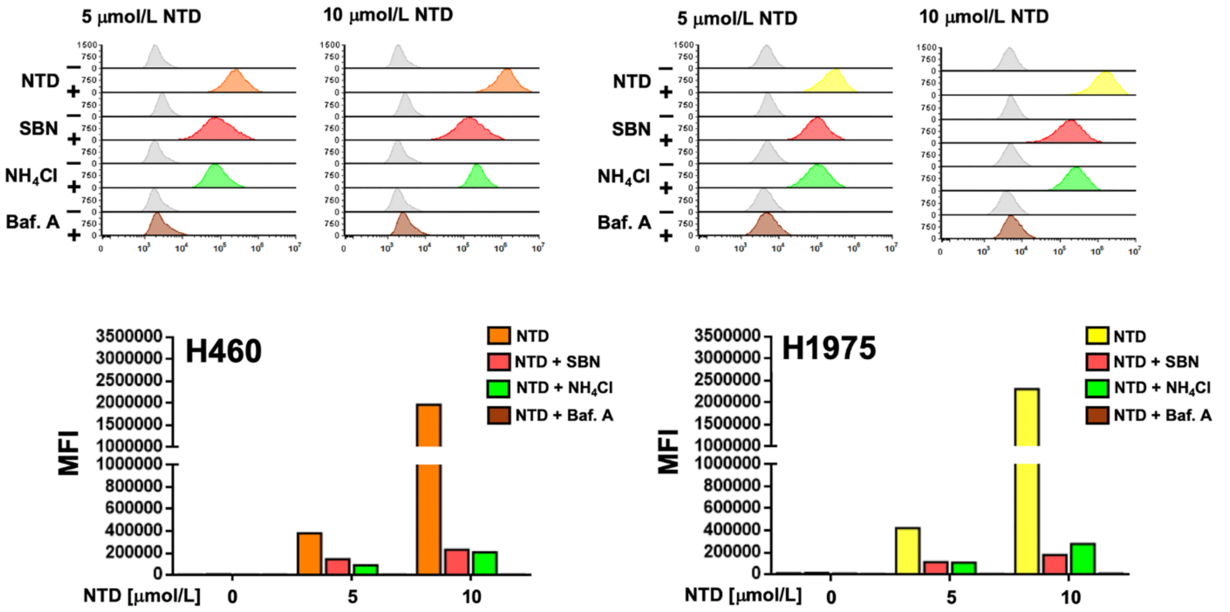

Figure S6. Comparative analysis of the effects of silibinin and alkalizing agents on the lysosomal sequestration of nintedanib in nintedanib-resistant NSCLC tumor cells. The impact of 100 μmol/L silibinin (SBN; 1 h pre-treatment), 10 mmol/L NH_4Cl, and 50 nmol/L bafilomycin A (Baf. A) on the intracellular accumulation of 5 and 10 μmol/L nintedanib (NTD) in H460 and H1975 cells was analyzed by flow cytometry (*top panels*) and live cell fluorescence microscopy (*bottom panels*) after 3 h drug exposure. Each experimental value represents the mean NTD-associated fluorescence (*columns*) ± S.D. (*bars*) of one representative experiment. (MFI: Mean Fluorescence Intensity).

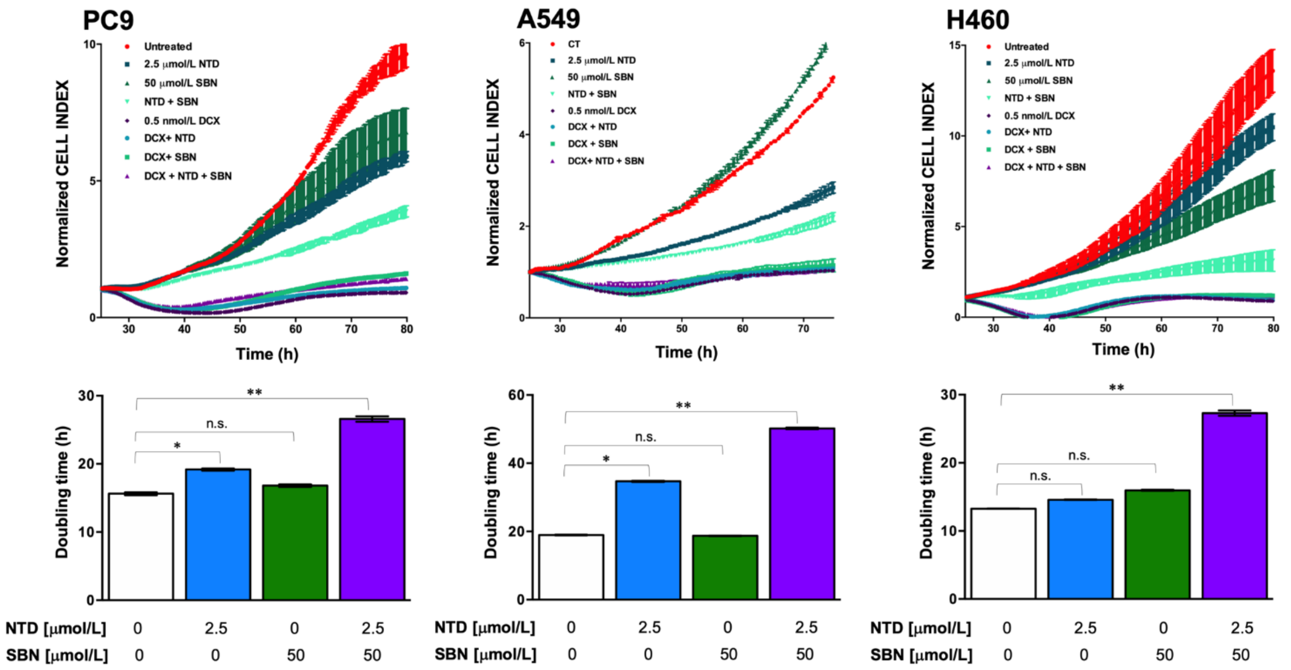

Figure S7. Real-time monitoring of cell proliferation in response to nintedanib, silibinin, and/or docetaxel. The rate of proliferation was monitored in real-time using the xCELLligence system. Figure shows the rates of proliferation (*top panels*) and cell doubling times (*bottom panels*) in the presence of nintedanib, silibinin, and/or docetaxel as determined by analyzing the growth curves shapes of PC-9, A549, and H460 cells between the 24 and 80 h hour interval. Doubling times results measured at 60 h are shown as mean (*columns*) ± SD (*error bars*) from at least two experiments in which triplicate wells were analyzed. Comparisons of means were performed by ANOVA. *P* values < 0.01 and < 0.001 were considered to be statistically significant (denoted as * and **, respectively; n.s. not significant).

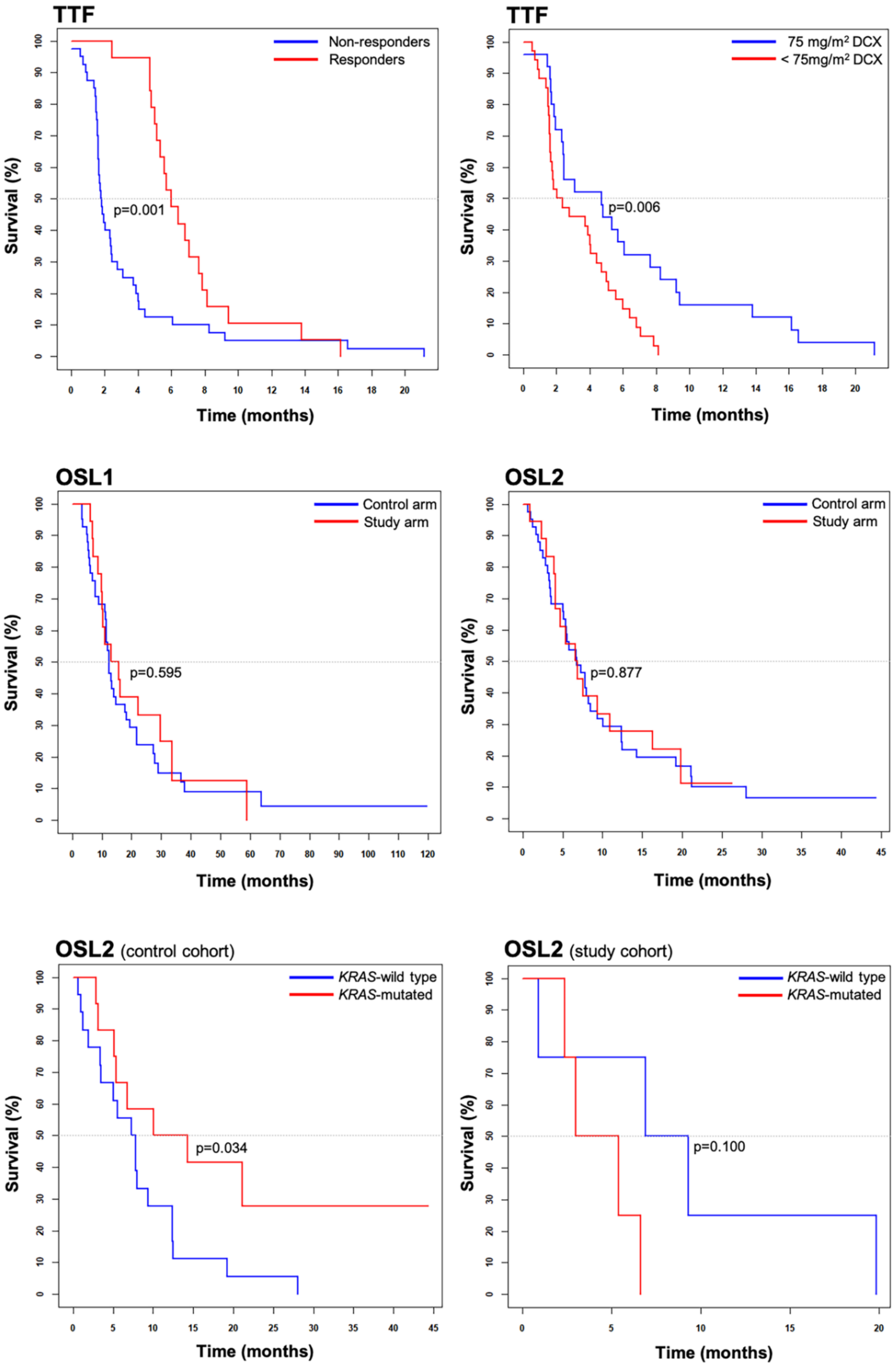

Figure S8. Impact of the silibinin-containing nutraceutical Legasil® on the clinical efficacy of Table 1. and OSL2 curves of patients stratified by non-responders/responders, 75 mg/m²/<75 mg/m² DCX, control arm/study arm, and KRAS mutational status.
